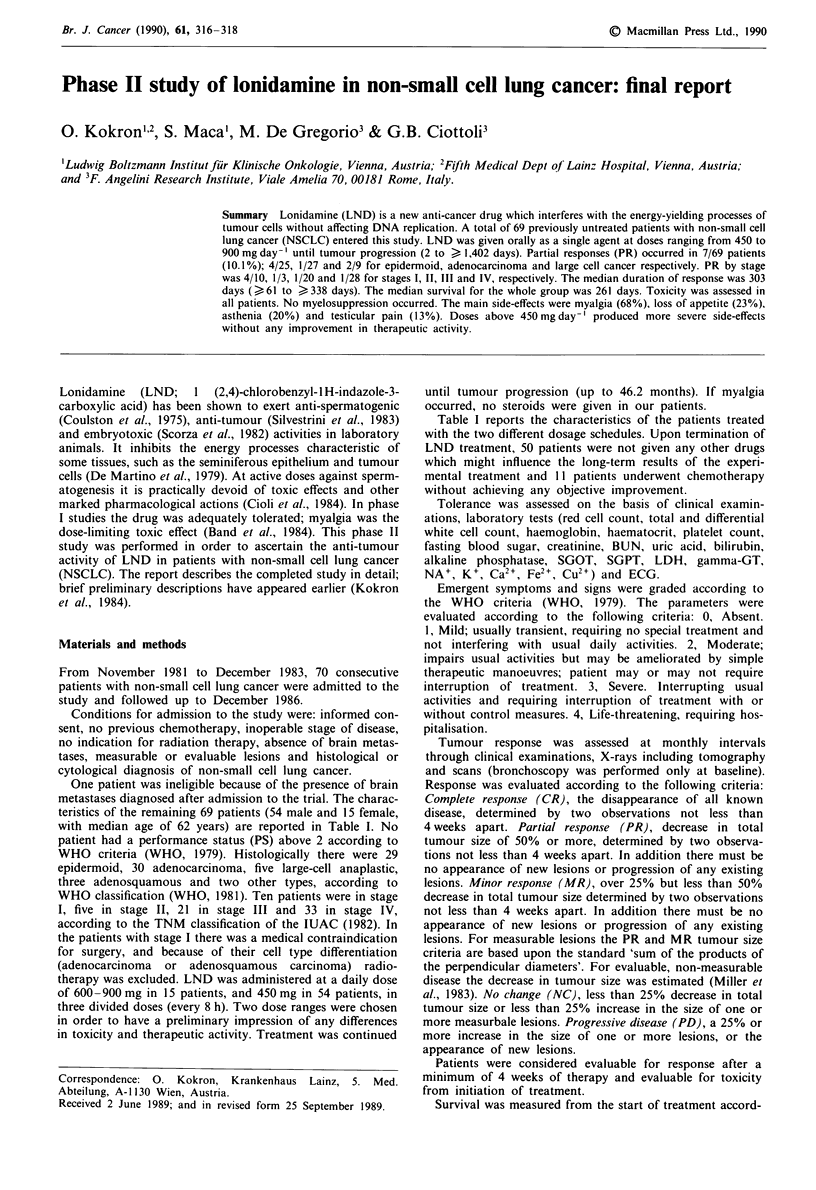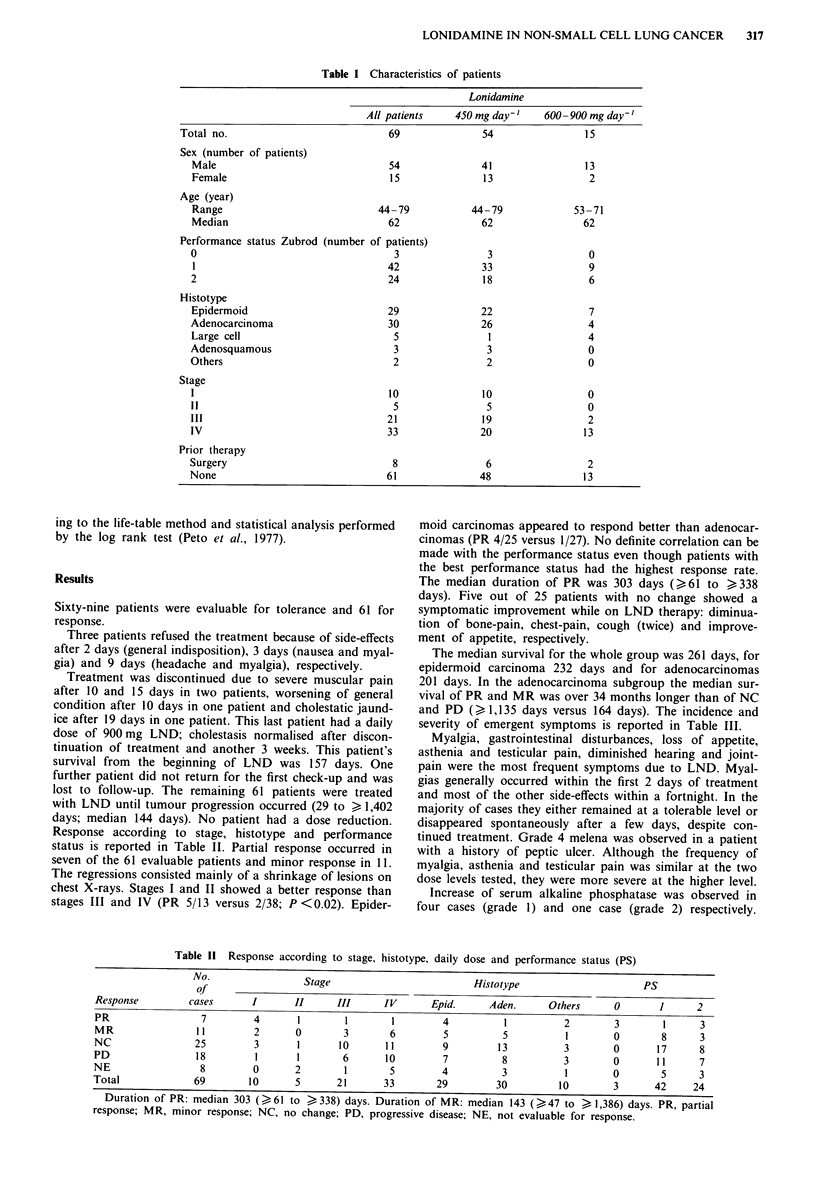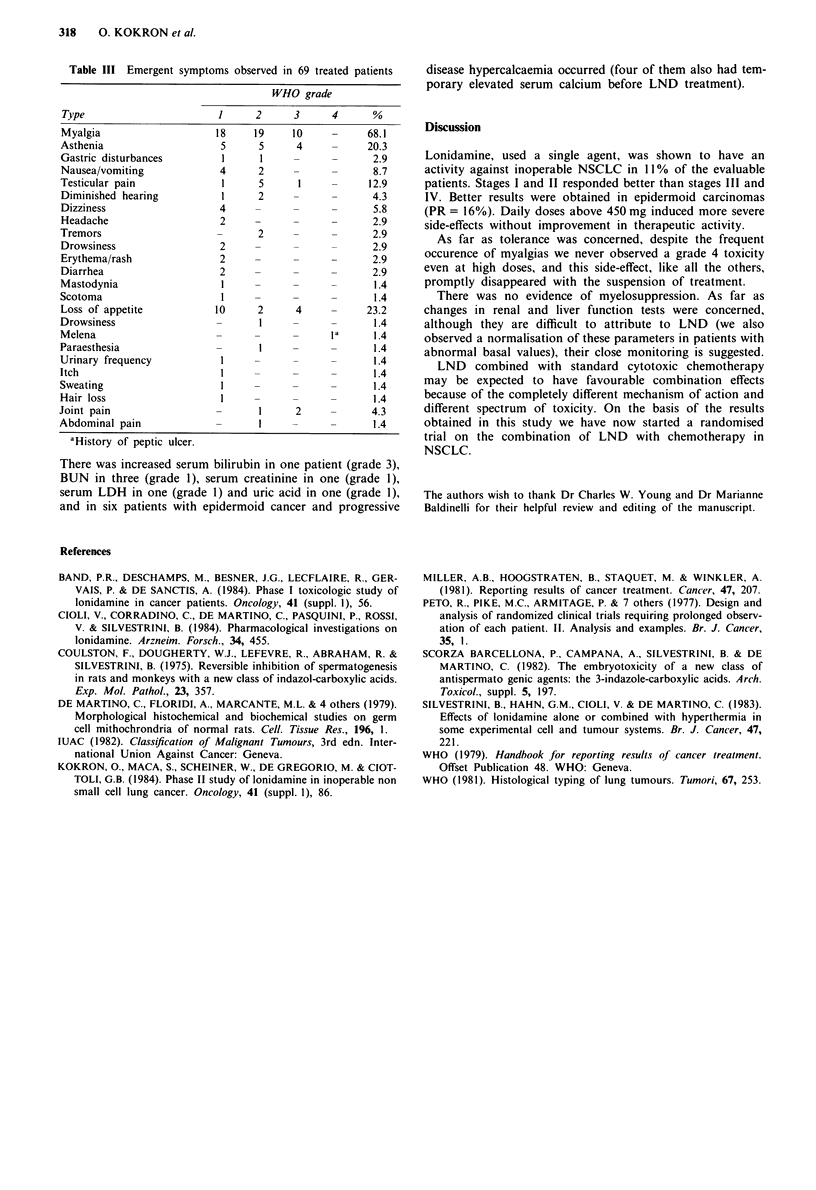# Phase II study of lonidamine in non-small cell lung cancer: final report.

**DOI:** 10.1038/bjc.1990.60

**Published:** 1990-02

**Authors:** O. Kokron, S. Maca, M. De Gregorio, G. B. Ciottoli

**Affiliations:** Ludwig Boltzmann Institut für Klinische Onkologie, Vienna, Austria.

## Abstract

Lonidamine (LND) is a new anti-cancer drug which interferes with the energy-yielding processes of tumour cells without affecting DNA replication. A total of 69 previously untreated patients with non-small cell lung cancer (NSCLC) entered this study. LND was given orally as a single agent at doses ranging from 450 to 900 mg day-1 until tumour progression (2 to greater than or equal to 1,402 days). Partial responses (PR) occurred in 7/69 patients (10.1%); 4/25, 1/27 and 2/9 for epidermoid, adenocarcinoma and large cell cancer respectively. PR by stage was 4/10, 1/3, 1/20 and 1/28 for stages I, II, III and IV, respectively. The median duration of response was 303 days (greater than or equal to 61 to greater than or equal to 338 days). The median survival for the whole group was 261 days. Toxicity was assessed in all patients. No myelosuppression occurred. The main side-effects were myalgia (68%), loss of appetite (23%), asthenia (20%) and testicular pain (13%). Doses above 450 mg day-1 produced more severe side-effects without any improvement in therapeutic activity.


Br. J. Cancer (1990), 61, 316-318 © Macmillan Press Ltd., 1990

### Phase II study of lonidamine in non-small cell lung cancer: final report

O. Kokron[1,2], S. Maca[1], M. De Gregorio[3] & G.B. Ciottoli[3]

[1]*Ludwig Boltzmann Institut für Klinische Onkologie, Vienna, Austria;* [2]*Fifth Medical Dept of Lainz Hospital, Vienna, Austria; and* [3]*F. Angelini Research Institute, Viale Amelia 70, 00181 Rome, Italy.*

**Summary** Lonidamine (LND) is a new anti-cancer drug which interferes with the energy-yielding processes of tumour cells without affecting DNA replication. A total of 69 previously untreated patients with non-small cell lung cancer (NSCLC) entered this study. LND was given orally as a single agent at doses ranging from 450 to 900 mg day$^{-1}$ until tumour progression (2 to $\geqslant$1,402 days). Partial responses (PR) occurred in 7/69 patients (10.1%); 4/25, 1/27 and 2/9 for epidermoid, adenocarcinoma and large cell cancer respectively. PR by stage was 4/10, 1/3, 1/20 and 1/28 for stages I, II, III and IV, respectively. The median duration of response was 303 days ($\geqslant$61 to $\geqslant$338 days). The median survival for the whole group was 261 days. Toxicity was assessed in all patients. No myelosuppression occurred. The main side-effects were myalgia (68%), loss of appetite (23%), asthenia (20%) and testicular pain (13%). Doses above 450 mg day$^{-1}$ produced more severe side-effects without any improvement in therapeutic activity.

Lonidamine (LND; 1 (2,4)-chlorobenzyl-1H-indazole-3-carboxylic acid) has been shown to exert anti-spermatogenic (Coulston *et al.*, 1975), anti-tumour (Silvestrini *et al.*, 1983) and embryotoxic (Scorza *et al.*, 1982) activities in laboratory animals. It inhibits the energy processes characteristic of some tissues, such as the seminiferous epithelium and tumour cells (De Martino *et al.*, 1979). At active doses against spermatogenesis it is practically devoid of toxic effects and other marked pharmacological actions (Cioli *et al.*, 1984). In phase I studies the drug was adequately tolerated; myalgia was the dose-limiting toxic effect (Band *et al.*, 1984). This phase II study was performed in order to ascertain the anti-tumour activity of LND in patients with non-small cell lung cancer (NSCLC). The report describes the completed study in detail; brief preliminary descriptions have appeared earlier (Kokron *et al.*, 1984).

### Materials and methods

From November 1981 to December 1983, 70 consecutive patients with non-small cell lung cancer were admitted to the study and followed up to December 1986.

Conditions for admission to the study were: informed consent, no previous chemotherapy, inoperable stage of disease, no indication for radiation therapy, absence of brain metastases, measurable or evaluable lesions and histological or cytological diagnosis of non-small cell lung cancer.

One patient was ineligible because of the presence of brain metastases diagnosed after admission to the trial. The characteristics of the remaining 69 patients (54 male and 15 female, with median age of 62 years) are reported in Table I. No patient had a performance status (PS) above 2 according to WHO criteria (WHO, 1979). Histologically there were 29 epidermoid, 30 adenocarcinoma, five large-cell anaplastic, three adenosquamous and two other types, according to WHO classification (WHO, 1981). Ten patients were in stage I, five in stage II, 21 in stage III and 33 in stage IV, according to the TNM classification of the IUAC (1982). In the patients with stage I there was a medical contraindication for surgery, and because of their cell type differentiation (adenocarcinoma or adenosquamous carcinoma) radiotherapy was excluded. LND was administered at a daily dose of 600-900 mg in 15 patients, and 450 mg in 54 patients, in three divided doses (every 8 h). Two dose ranges were chosen in order to have a preliminary impression of any differences in toxicity and therapeutic activity. Treatment was continued until tumour progression (up to 46.2 months). If myalgia occurred, no steroids were given in our patients.

Table I reports the characteristics of the patients treated with the two different dosage schedules. Upon termination of LND treatment, 50 patients were not given any other drugs which might influence the long-term results of the experimental treatment and 11 patients underwent chemotherapy without achieving any objective improvement.

Tolerance was assessed on the basis of clinical examinations, laboratory tests (red cell count, total and differential white cell count, haemoglobin, haematocrit, platelet count, fasting blood sugar, creatinine, BUN, uric acid, bilirubin, alkaline phosphatase, SGOT, SGPT, LDH, gamma-GT, $NA^+$, $K^+$, $Ca^{2+}$, $Fe^{2+}$, $Cu^{2+}$) and ECG.

Emergent symptoms and signs were graded according to the WHO criteria (WHO, 1979). The parameters were evaluated according to the following criteria: 0, Absent. 1, Mild; usually transient, requiring no special treatment and not interfering with usual daily activities. 2, Moderate; impairs usual activities but may be ameliorated by simple therapeutic manoeuvres; patient may or may not require interruption of treatment. 3, Severe. Interrupting usual activities and requiring interruption of treatment with or without control measures. 4, Life-threatening, requiring hospitalisation.

Tumour response was assessed at monthly intervals through clinical examinations, X-rays including tomography and scans (bronchoscopy was performed only at baseline). Response was evaluated according to the following criteria: *Complete response (CR)*, the disappearance of all known disease, determined by two observations not less than 4 weeks apart. *Partial response (PR)*, decrease in total tumour size of 50% or more, determined by two observations not less than 4 weeks apart. In addition there must be no appearance of new lesions or progression of any existing lesions. *Minor response (MR)*, over 25% but less than 50% decrease in total tumour size determined by two observations not less than 4 weeks apart. In addition there must be no appearance of new lesions or progression of any existing lesions. For measurable lesions the PR and MR tumour size criteria are based upon the standard 'sum of the products of the perpendicular diameters'. For evaluable, non-measurable disease the decrease in tumour size was estimated (Miller *et al.*, 1983). *No change (NC)*, less than 25% decrease in total tumour size or less than 25% increase in the size of one or more measurbale lesions. *Progressive disease (PD)*, a 25% or more increase in the size of one or more lesions, or the appearance of new lesions.

Patients were considered evaluable for response after a minimum of 4 weeks of therapy and evaluable for toxicity from initiation of treatment.

Survival was measured from the start of treatment accord-

Correspondence: O. Kokron, Krankenhaus Lainz, 5. Med. Abteilung, A-1130 Wien, Austria.
Received 2 June 1989; and in revised form 25 September 1989.

**Table I** Characteristics of patients

| | Lonidamine | | |
|---|---|---|---|
| | *All patients* | *450 mg day⁻¹* | *600–900 mg day⁻¹* |
| Total no. | 69 | 54 | 15 |
| Sex (number of patients) | | | |
| Male | 54 | 41 | 13 |
| Female | 15 | 13 | 2 |
| Age (year) | | | |
| Range | 44–79 | 44–79 | 53–71 |
| Median | 62 | 62 | 62 |
| Performance status Zubrod (number of patients) | | | |
| 0 | 3 | 3 | 0 |
| 1 | 42 | 33 | 9 |
| 2 | 24 | 18 | 6 |
| Histotype | | | |
| Epidermoid | 29 | 22 | 7 |
| Adenocarcinoma | 30 | 26 | 4 |
| Large cell | 5 | 1 | 4 |
| Adenosquamous | 3 | 3 | 0 |
| Others | 2 | 2 | 0 |
| Stage | | | |
| I | 10 | 10 | 0 |
| II | 5 | 5 | 0 |
| III | 21 | 19 | 2 |
| IV | 33 | 20 | 13 |
| Prior therapy | | | |
| Surgery | 8 | 6 | 2 |
| None | 61 | 48 | 13 |

ing to the life-table method and statistical analysis performed by the log rank test (Peto *et al.*, 1977).

### Results

Sixty-nine patients were evaluable for tolerance and 61 for response.

Three patients refused the treatment because of side-effects after 2 days (general indisposition), 3 days (nausea and myalgia) and 9 days (headache and myalgia), respectively.

Treatment was discontinued due to severe muscular pain after 10 and 15 days in two patients, worsening of general condition after 10 days in one patient and cholestatic jaundice after 19 days in one patient. This last patient had a daily dose of 900 mg LND; cholestasis normalised after discontinuation of treatment and another 3 weeks. This patient's survival from the beginning of LND was 157 days. One further patient did not return for the first check-up and was lost to follow-up. The remaining 61 patients were treated with LND until tumour progression occurred (29 to $\geqslant$1,402 days; median 144 days). No patient had a dose reduction. Response according to stage, histotype and performance status is reported in Table II. Partial response occurred in seven of the 61 evaluable patients and minor response in 11. The regressions consisted mainly of a shrinkage of lesions on chest X-rays. Stages I and II showed a better response than stages III and IV (PR 5/13 versus 2/38; $P<0.02$). Epidermoid carcinomas appeared to respond better than adenocarcinomas (PR 4/25 versus 1/27). No definite correlation can be made with the performance status even though patients with the best performance status had the highest response rate. The median duration of PR was 303 days ($\geqslant$61 to $\geqslant$338 days). Five out of 25 patients with no change showed a symptomatic improvement while on LND therapy: diminuation of bone-pain, chest-pain, cough (twice) and improvement of appetite, respectively.

The median survival for the whole group was 261 days, for epidermoid carcinoma 232 days and for adenocarcinomas 201 days. In the adenocarcinoma subgroup the median survival of PR and MR was over 34 months longer than of NC and PD ($\geqslant$1,135 days versus 164 days). The incidence and severity of emergent symptoms is reported in Table III.

Myalgia, gastrointestinal disturbances, loss of appetite, asthenia and testicular pain, diminished hearing and joint-pain were the most frequent symptoms due to LND. Myalgias generally occurred within the first 2 days of treatment and most of the other side-effects within a fortnight. In the majority of cases they either remained at a tolerable level or disappeared spontaneously after a few days, despite continued treatment. Grade 4 melena was observed in a patient with a history of peptic ulcer. Although the frequency of myalgia, asthenia and testicular pain was similar at the two dose levels tested, they were more severe at the higher level.

Increase of serum alkaline phosphatase was observed in four cases (grade 1) and one case (grade 2) respectively.

**Table II** Response according to stage, histotype, daily dose and performance status (PS)

| *Response* | *No. of cases* | *Stage* | | | | *Histotype* | | | *PS* | | |
|---|---|---|---|---|---|---|---|---|---|---|---|
| | | *I* | *II* | *III* | *IV* | *Epid.* | *Aden.* | *Others* | *0* | *1* | *2* |
| PR | 7 | 4 | 1 | 1 | 1 | 4 | 1 | 2 | 3 | 1 | 3 |
| MR | 11 | 2 | 0 | 3 | 6 | 5 | 5 | 1 | 0 | 8 | 3 |
| NC | 25 | 3 | 1 | 10 | 11 | 9 | 13 | 3 | 0 | 17 | 8 |
| PD | 18 | 1 | 1 | 6 | 10 | 7 | 8 | 3 | 0 | 11 | 7 |
| NE | 8 | 0 | 2 | 1 | 5 | 4 | 3 | 1 | 0 | 5 | 3 |
| Total | 69 | 10 | 5 | 21 | 33 | 29 | 30 | 10 | 3 | 42 | 24 |

Duration of PR: median 303 ($\geqslant$61 to $\geqslant$338) days. Duration of MR: median 143 ($\geqslant$47 to $\geqslant$1,386) days. PR, partial response; MR, minor response; NC, no change; PD, progressive disease; NE, not evaluable for response.

**Table III** Emergent symptoms observed in 69 treated patients

| | WHO grade | | | | |
|---|---|---|---|---|---|
| *Type* | *1* | *2* | *3* | *4* | *%* |
| Myalgia | 18 | 19 | 10 | – | 68.1 |
| Asthenia | 5 | 5 | 4 | – | 20.3 |
| Gastric disturbances | 1 | 1 | – | – | 2.9 |
| Nausea/vomiting | 4 | 2 | – | – | 8.7 |
| Testicular pain | 1 | 5 | 1 | – | 12.9 |
| Diminished hearing | 1 | 2 | – | – | 4.3 |
| Dizziness | 4 | – | – | – | 5.8 |
| Headache | 2 | – | – | – | 2.9 |
| Tremors | – | 2 | – | – | 2.9 |
| Drowsiness | 2 | – | – | – | 2.9 |
| Erythema/rash | 2 | – | – | – | 2.9 |
| Diarrhea | 2 | – | – | – | 2.9 |
| Mastodynia | 1 | – | – | – | 1.4 |
| Scotoma | 1 | – | – | – | 1.4 |
| Loss of appetite | 10 | 2 | 4 | – | 23.2 |
| Drowsiness | – | 1 | – | – | 1.4 |
| Melena | – | – | – | 1[a] | 1.4 |
| Paraesthesia | – | 1 | – | – | 1.4 |
| Urinary frequency | 1 | – | – | – | 1.4 |
| Itch | 1 | – | – | – | 1.4 |
| Sweating | 1 | – | – | – | 1.4 |
| Hair loss | 1 | – | – | – | 1.4 |
| Joint pain | – | 1 | 2 | – | 4.3 |
| Abdominal pain | – | 1 | – | – | 1.4 |

[a]History of peptic ulcer.

There was increased serum bilirubin in one patient (grade 3), BUN in three (grade 1), serum creatinine in one (grade 1), serum LDH in one (grade 1) and uric acid in one (grade 1), and in six patients with epidermoid cancer and progressive disease hypercalcaemia occurred (four of them also had temporary elevated serum calcium before LND treatment).

### Discussion

Lonidamine, used a single agent, was shown to have an activity against inoperable NSCLC in 11% of the evaluable patients. Stages I and II responded better than stages III and IV. Better results were obtained in epidermoid carcinomas (PR = 16%). Daily doses above 450 mg induced more severe side-effects without improvement in therapeutic activity.

As far as tolerance was concerned, despite the frequent occurence of myalgias we never observed a grade 4 toxicity even at high doses, and this side-effect, like all the others, promptly disappeared with the suspension of treatment.

There was no evidence of myelosuppression. As far as changes in renal and liver function tests were concerned, although they are difficult to attribute to LND (we also observed a normalisation of these parameters in patients with abnormal basal values), their close monitoring is suggested.

LND combined with standard cytotoxic chemotherapy may be expected to have favourable combination effects because of the completely different mechanism of action and different spectrum of toxicity. On the basis of the results obtained in this study we have now started a randomised trial on the combination of LND with chemotherapy in NSCLC.

The authors wish to thank Dr Charles W. Young and Dr Marianne Baldinelli for their helpful review and editing of the manuscript.

### References

BAND, P.R., DESCHAMPS, M., BESNER, J.G., LECFLAIRE, R., GERVAIS, P. & DE SANCTIS, A. (1984). Phase I toxicologic study of lonidamine in cancer patients. *Oncology*, **41** (suppl. 1), 56.

CIOLI, V., CORRADINO, C., DE MARTINO, C., PASQUINI, P., ROSSI, V. & SILVESTRINI, B. (1984). Pharmacological investigations on lonidamine. *Arzneim. Forsch.*, **34**, 455.

COULSTON, F., DOUGHERTY, W.J., LEFEVRE, R., ABRAHAM, R. & SILVESTRINI, B. (1975). Reversible inhibition of spermatogenesis in rats and monkeys with a new class of indazol-carboxylic acids. *Exp. Mol. Pathol.*, **23**, 357.

DE MARTINO, C., FLORIDI, A., MARCANTE, M.L. & 4 others (1979). Morphological histochemical and biochemical studies on germ cell mithochrondria of normal rats. *Cell. Tissue Res.*, **196**, 1.

IUAC (1982). *Classification of Malignant Tumours*, 3rd edn. International Union Against Cancer: Geneva.

KOKRON, O., MACA, S., SCHEINER, W., DE GREGORIO, M. & CIOTTOLI, G.B. (1984). Phase II study of lonidamine in inoperable non small cell lung cancer. *Oncology*, **41** (suppl. 1), 86.

MILLER, A.B., HOOGSTRATEN, B., STAQUET, M. & WINKLER, A. (1981). Reporting results of cancer treatment. *Cancer*, **47**, 207.

PETO, R., PIKE, M.C., ARMITAGE, P. & 7 others (1977). Design and analysis of randomized clinical trials requiring prolonged observation of each patient. II. Analysis and examples. *Br. J. Cancer*, **35**, 1.

SCORZA BARCELLONA, P., CAMPANA, A., SILVESTRINI, B. & DE MARTINO, C. (1982). The embryotoxicity of a new class of antispermato genic agents: the 3-indazole-carboxylic acids. *Arch. Toxicol.*, suppl. **5**, 197.

SILVESTRINI, B., HAHN, G.M., CIOLI, V. & DE MARTINO, C. (1983). Effects of lonidamine alone or combined with hyperthermia in some experimental cell and tumour systems. *Br. J. Cancer*, **47**, 221.

WHO (1979). *Handbook for reporting results of cancer treatment*. Offset Publication 48. WHO: Geneva.

WHO (1981). Histological typing of lung tumours. *Tumori*, **67**, 253.